# *Éxito!*: Making an Impact in Training Latinos for Doctorates and Cancer Research

**DOI:** 10.1007/s13187-018-1397-6

**Published:** 2018-07-16

**Authors:** Amelie G. Ramirez, Kipling J. Gallion, Arely Perez, Rebecca T. Adeigbe, Edgar Munoz, Rena J. Pasick

**Affiliations:** 1 UT Health San Antonio, School of Medicine, Department of Epidemiology and Biostatistics, Institute for Health Promotion Research, 7411 John Smith Dr., Suite 1000, San Antonio, TX 78229 USA; 2 University of California, San Francisco, Division of General Internal Medicine, Helen Diller Family Comprehensive Cancer Center, Box 0128, 1450 3rd Street HD 553, San Francisco, CA 94143 USA

**Keywords:** Cancer disparities, Training, Latinos

## Abstract

Latinos lag behind other racial/ethnic groups in pursuit of master’s and doctoral degrees in public health and the health sciences. *Éxito!* is modeled after the Minority Training Program in Cancer Control Research (MTPCCR), which found that Latino participants went on to doctoral programs at a lower rate (12%) than African American (36%) and Asian participants (33%)*. Éxito!* Latino Cancer Research Leadership Training is designed to increase the number of Latinos who pursue doctoral degrees and careers in cancer health disparity (CHD) research. The program has three components: recruitment with partnering universities and associations, an ethnically tailored intensive 5-day summer institute (SI), and 6-month paid internships offered on a competitive basis. Up to 20 master’s level students/master’s level health professionals are selected annually to participate in the SI; faculty are leaders in Latino CHD research. Funded by the National Cancer Institute (NCI) from 2011 to 2015, *Éxito!* recruited 101 summer institute participants and awarded 21 internships. Analyses of pre- and post-institute surveys showed significant increases in confidence to apply to a doctoral program and academic self-efficacy among summer institute participants, and significantly increased research skills among interns. Forty-three percent of *Éxito!* program alumni applied to a doctoral program (our main outcome) and 29.7% were currently enrolled. This is nearly double the rate for MTPCCR Latino participants (17%) for the corresponding time period. *Éxito!* is a model pipeline program for encouragement of Latinos on to doctoral programs (e.g., PhD and DrPH) with the potential to increase the pool of cancer health disparity researchers.

## Introduction

Cancer has surpassed cardiovascular disease as the leading cause of death for Latinos in the USA [1]. Moreover, an estimated 142% increase in cancer diagnoses among Latinos will occur between 2010 and 2030 [2]. Yet, the field of cancer control research, composed of many public health and health science disciplines, continues to lack ethnic diversity, especially Latino representation [3]. There is a substantial lack of “insider researchers” who share cultural characteristics with the communities who experience disparities and serve as critical players in helping reduce cancer health disparities [4–6]. The need of these “insider researchers” is four fold: insider researchers have a deeper and more culturally attuned understanding of the health disparities experienced by their own community; they dedicate themselves more passionately and personally than any other group to the elimination of these disparities; they engender trust and a high degree of credibility among community members and leaders whose partnership is essential to truly relevant, actionable research, and they serve as role models and mentors for future generations of researchers [7–9].

Although there are growing numbers of Latinos at the undergraduate level, they lag behind Whites and other racial/ethnic groups in pursuit of master’s degrees and doctoral degrees in public health and the health sciences [10, 11]. Latinos make up 17% of the US population [12], but in 2010–2011 when our program began, only 7.5% of master’s and doctoral degrees (combined) were earned by Latinos at US schools of public health, compared to 59.2% for Whites [10]. This disparity may be caused by unwelcoming campus climates, absence of diversity among faculty and other personnel at institutions, and financial constraints or family obligations [13, 14].

## Background

Because the literature on educational pipeline diversity reveals that there may be unique challenges that impede pursuit of doctoral-level education by Latinos [15–17], and to our knowledge, there are no national pipeline programs for Latinos in public health and social/behavioral research, the *Éxito! Latino Cancer Research Leadership Training* (*Éxito!*) program was created by the Institute for Health Promotion Research at UT Health San Antonio with funding from the National Cancer Institute (NCI). *Éxito!* conducts an annual 5-day summer institute and offers 6-month internships to encourage Latino master’s level students and graduates to pursue a doctoral degree and a career in cancer control research, with a focus on reduction of Latino cancer health disparities. *Éxito!* is modeled after the successful Minority Training Program in Cancer Control Research (MTPCCR) with sites at the University of California, San Francisco (UCSF) and UCLA, which seeks to increase diversity in public health and social/behavioral doctoral programs and cancer disparity research [3, 18, 19]. A pivotal impetus for *Éxito!* was the lower rate at which MTPCCR’s Latino participants went on to doctoral programs (12%) compared with African American (36%) and Asian participants (33%) [3].

The long-term goal of *Éxito!* is to increase the number of Latinos who pursue doctoral degrees and careers in cancer control research focusing on the causes and solutions to Latino cancer health disparities. On an ongoing basis, we monitor the number of participants who apply and are accepted into a doctoral program. We also assess intermediate impacts that include increased confidence to apply to a doctoral program in the next year or within 5 years, increased participants’ confidence that they have the skills to successfully apply to a doctoral program, and increased number of participants who indicate that they will pursue a career in Latino cancer health disparity research.

The purpose of this article is to describe the impact of the first 5 years (2011–2015) of *Éxito!* as indicated by: (1) the number of individuals who participated in the program; (2) the influence of the summer institute and internships on participants’ confidence to pursue doctoral degrees and careers in cancer research; and (3) the number of participants who applied, were accepted, and are currently enrolled in doctoral programs.

## Theoretical Foundation of *Éxito!*

**Fig. 1:**
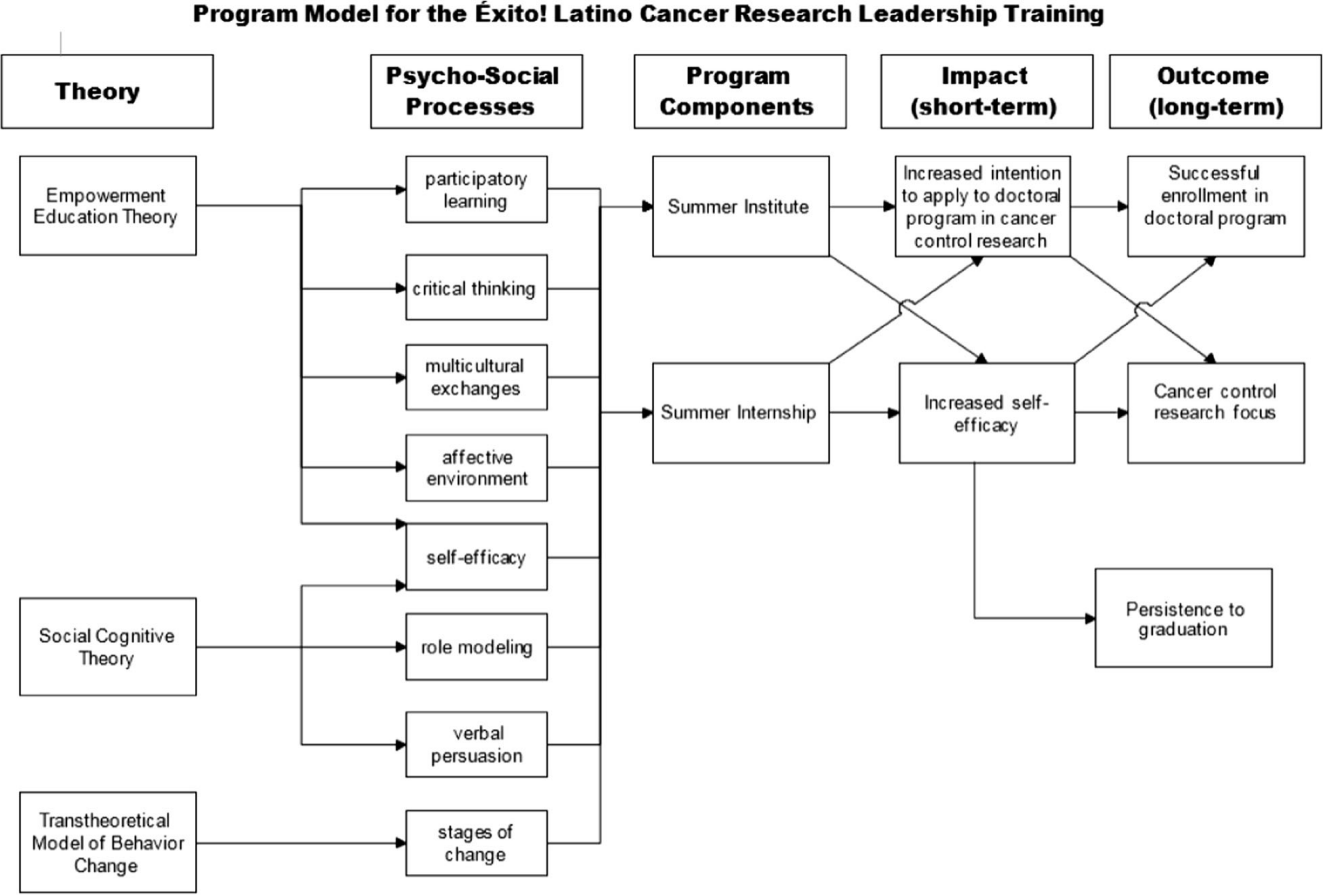
Model for *Exito*!. Program components flow from the conceptual framework. Intention to apply and academic self-efficacy are interim indicators toward the long-term outcomes of doctoral program enrollment and a research focus of cancer control

The structure and content of the *Éxito!* program were influenced by three models of behavior change (Fig. 1): (1) Freire’s empowerment education theory (EET) [20], (2) Bandura’s social cognitive theory (SCT) [21], and (3) the transtheoretical model of behavior change (TTM) [22]. These models were chosen to guide us in overcoming barriers to Latino doctoral level education and thus adequate representation among leaders in public health and health disparity research.

EET posits that the act of learning and ultimately educational attainment can empower students if instructors and mentors structure and engage in mutually created dialog for academic and social issues most relevant to the student’s lived experiences. Throughout the *Éxito!* 5-day summer institute, we sought to raise participants’ critical consciousness through problem-posing and solution-building dialog using examples applicable to their own experiences (see curriculum below). Four EET elements were used in the summer institute: (1) participatory presentations and group exercises; (2) critical thinking that engaged participants in reasoning as future researchers; (3) multicultural exchanges in which the group addressed various racial, ethnic, regional, age-based, and sexual differences based on personal and shared experiences; and (4) cultivation of an affective environment where participants and staff found acceptance and comfort through discussion of ethnic similarities and differences and other features, such as ethnic food and music. These exercises helped students understand how to more effectively communicate their specific needs and desires in varied academic climates including those that may initially be perceived as unsupportive.

Within the context of the ETT, SCT constructs were also applied to address behaviors resulting from individual, social, and environmental interactions. Three constructs from SCT had particular relevance to *Éxito!*: (1) perceived self-efficacy, (2) role modeling, and (3) positive verbal persuasion. These three constructs were employed to design a program that would help improve participants’ perceived confidence in their ability to successfully apply to graduate school, be accepted, and complete a doctoral degree. We created many opportunities to enhance self-efficacy by emphasizing specific information relevant to Latinos in grant writing and the funding mechanisms available to them. We also chose Latino role models to present their research and to discuss applying for funding. According to SCT, students should best be able to identify with researchers who have been “down the same road.”

Lastly, TTM, also known as the “stages of change” model, was used to identify each participant’s current level of readiness to apply to a doctoral program and to pursue a career in cancer control research. For example, some *Éxito!* program applicants were in the “contemplation” stage, meaning they were considering a doctoral education but had not taken action; others were in “pre-contemplation.” These individuals applied to our program not because they were interested in a doctorate but because a friend or advisor said the program would be a good opportunity. Consistent with the TTM, *Éxito!* engaged participants at their stage level by providing dialog, testimony, and constant “can do” encouragement to move the students to the action stage.

## *Éxito!* Program Components

### Participant Recruitment

Each year, *Éxito!* sought to enroll 20 master’s level students and professionals to attend a 5-day summer institute. Program recruitment materials were emailed to 50 universities, 240 faculty members, and 40 student groups during annual recruitment (November–February). The online applications included a personal statement, two recommendation letters, and master’s program academic transcript. Applicants were eligible if they self-identified as Latino or had experience working in Latino communities. In order to recruit participants who are qualified to apply and gain acceptance into doctoral programs, our eligibility criteria also included: (1) graduate school grades (minimum “B” average), (2) strong favorable institutional faculty recommendations, and (3) a written statement describing individual goals and expectations aligning with the program. The review process was conducted by *Éxito!* team members. For individuals accepted into the program, travel, lodging, and meals were supported by grant funds.

### Summer Institute

The summer institute curriculum addressed five overarching learning objectives: (1) gain exposure to the need and opportunities for Latino researchers in cancer control and cancer health disparity research; (2) gain knowledge about the importance of cancer control research and the potential to reduce health disparities and improve cancer-related health outcomes among Latinos; (3) interact and establish networks with accomplished researchers from diverse Latino backgrounds and disciplines; (4) obtain an overview of Latino health disparity research disciplines related to cancer control research (i.e., epidemiology, behavioral research, surveillance, survivorship); and (5) comprehend and use necessary skills, resources, and support to apply to a doctoral program. The summer institute curriculum consisted of the following themes and activities:

Day 1, *What’s Going on in Health Disparities, Including Cancer Control Research, and Why We (Latinos) Are Here*, examined participants’ culture and their personal stories while also piquing their interest in cancer research. Each person was asked to bring a cultural object that represents their background and to describe it as part of their self-introduction. This made the process far more personal than introductions typically encountered in academic gatherings, and proved to be highly emotional at times as participants were invited to “bring their ancestors into the room.” This evokes the pyschosocial processes of the EET by cultivating an environment of acceptance and comfort through discussion of ethnic similarities and differences. Day 2, *Reaching the “Hard-to-Reach”: The Range & Reach of Health Disparities and Cancer Control*, presented participants with the most recent information on cancer prevalence and health disparities among Latinos as well as the range of opportunities in cancer research from clinical to survivorship research. Presenters were role model (Latino) researchers, specifically so that participants could relate closely to them. Day 3, *Why Culture Matters & Tools of the Trade*, targeted the social and cultural differences experienced by Latinos that lead to disparities in cancer prevention, diagnosis, and treatment. Participants heard from *Éxito!* alumni enrolled in doctoral programs and received tips and tools for applying to and completing a doctoral program. In addition, participants received a 2-h writing workshop by a writing specialist. Day 4, *Hear from the Experts: How to Apply to Doctoral Programs* provided resources needed to apply to, get accepted to, and thrive in a doctoral program. Participants also heard from faculty researchers and graduate program admissions personnel. Day 5, *Stepping Out: Transition from Student to Researcher*, tied everything together. Participants heard from successful Latino faculty including PhDs/DrPHs and physicians on the importance of networking, self-representation, and the skills needed to effectively achieve their educational and career goals.

Throughout the summer institute, Latino researchers discussed their personal journeys to and through the doctorate and described their current career roles and work environments. Participants took part in interactive group discussions to foster networking and sharing of personal experiences to better prepare for the cultural challenges that come with doctoral work. Sessions of sharing research and career aspirations promoted “thinking like a researcher” among participants, and others facilitated important dialog of what it means to be a minority in an academic environment and the importance of work-life balance for minority academia as well. All these highly participatory interactive sessions enabled understanding of cultural experiences among participants and encouraged interpersonal bonding.

### Part 3, *Éxito!* Internships

Each year, the program awarded up to six 6-month internships to *Éxito!* alumni and new participants. The internships were designed to: (1) expose program participants/alumni to Latino cancer health disparity research, (2) provide valuable research experience, and (3) enhance research skills. Intern and mentor stipends were funded by the *Éxito!* grant. To be considered for an internship, applicants were asked to include a work plan stating each individual’s internship goals and objectives. All internships were conducted at the applicant’s home institution or university, and interns identified a mentor as part of the work plan. Interns were required to complete monthly progress reports outlining accomplishments and lessons learned or obstacles faced. These reports helped interns make the most of their research experience and aided mentors in guiding them as they moved through the TTM stages of change.

## Methods

The degree to which the *Éxito!* Program met its goals and objectives between 2010 and 2015 was evaluated using process and outcome evaluation.

### Process Evaluation

The *Éxito!* process evaluation provided a record of activity and gave immediate feedback on program design and implementation.

#### Part 1, Participant Recruitment

Process measures included: (1) number of Latino applicants to the summer institute, (2) participant ratings of each summer institute session as indicated in daily surveys, (3) number of participants who initiated and completed internships, and (4) ratio of applicants to available slots in the summer institute.

#### Part 2, Summer Institute

Surveys used to evaluate the *Éxito!* program were originally developed by the MTPCCR team, who conducted focus groups with minority master’s students at California State University, Hayward, California State University, Northridge, San Francisco State University, and UCLA. The resulting surveys had the following domains: family immigration and education history, ethnic identity and acculturative stress, perceived discrimination and general academic influences and preparation experience with academic enrichment programs, motivators and barriers to pursuit of doctoral training and academic self-efficacy scale.

Based on the formative research conducted by the MTPCCR program, the *Éxito!* program surveys were replicated and used to measure participants’ self-assessments regarding capability to pursue a doctoral degree and perception of having the necessary skills to achieve this goal. More specifically, the summer institute surveys measured participants’ perception of their capability to overcome acculturative, language, financial, family, academic, cultural, and personal barriers to doctoral application and enrollment. Initial instruments were modified after the first year of the program.

Process measures for the summer institute included daily evaluations with both quantitative ratings and open-ended questions. Participants were asked to rate the content and quality of each presentation and the day as a whole using a five-point Likert-type scale from 1 = “poor” to 5 = “excellent”. Participants were also asked to complete open-ended questions indicating the most and least useful components of the day. Additionally, as part of the daily evaluations, participants were asked to evaluate the presenters, activities, and overall logistics (program length, pace). Feedback received on the daily evaluations were used for continual process improvement for subsequent days of the summer institute and future summer institute planning. Average daily ratings were compiled to track achievement of our aim to maintain overall ratings at the very good to excellent level.

#### Part 3, *Éxito!* Internships

Internship quality was assessed by regular meetings with interns and program staff to debrief and trouble-shoot as needed, by intern final reports, and using brief pre- and post-internship surveys. Mentors were also required to complete a survey at the conclusion of the internship.

### Impact Evaluation

A REDCap online database was developed to track alumni, their doctoral applications, and all survey responses. Developing a database was essential because monitoring progress toward the program’s goal of doctoral program enrollment and work in cancer control required both short- and long-term monitoring.

#### Part 2, Summer Institute

Interim progress toward our main goal was measured by assessing changes in academic self-efficacy and intentions to apply to a doctoral program and pursue a career in cancer control research. This information came from participant responses to pre- and post-summer institute surveys. Prior to the summer institute, academic self-efficacy was measured using an academic self-efficacy scale, and participant intention to seek a doctoral degree was measured using a five-point scale (5 = “very confident I will apply” and 1 = “very confident I will not apply”). Similarly, participants were asked about their confidence in pursuing a career in Latino cancer health disparity research using the same five-point scale. A post-institute survey then assessed changes in both impacts.

#### Part 3, *Éxito!* Internships

Every year, interns were required to complete a pre- and post-internship survey to determine change in research skills as a result of their *Éxito!* internship. The survey was focused on academic and research readiness and asked participants to respond the statement “Please rate yourself on the following skills, specifically as they pertain to your readiness for a doctoral program.” The skills listed were: writing skills, time management skills, computer skills, statistical research skills, qualitative research skills, conceptual skills, and interpersonal skills. Participants could rate their skills as either excellent, very good, good, fair, or poor. Additionally, to determine if the internships had an influence on participants, interns’ confidence in pursuing a doctoral degree and career in Latino cancer health disparities was reassessed as part of the post-internship survey. Mentors were also required to complete a post-internship survey and reflect on their interns’ research skills and perceived motivation to pursue a doctoral degree and a career in cancer research.

## Outcome Evaluation

### Annual Alumni Survey

*Éxito!’s* main outcomes are applications to and acceptance into doctoral programs. This information is collected every year using our annual alumni survey and through ad hoc reports sent by alumni. The annual alumni survey also collects alumni’s thoughts regarding their summer institute experience by asking questions such as, “How much did the *Éxito!* program affect your interest in cancer-related research?” In addition, the survey captures alumni’s current education status, status regarding doctoral program applications and acceptances, and intention to apply (for those who have not done so). Participants were also asked about their intention to pursue a career directly (e.g., tobacco control, mammography, cancer patient navigation) or indirectly (promotion of fitness or nutrition, end of life care, health literacy) related to cancer research. Cancer focus among master’s level and doctoral students currently employed is measured as well; program staff assess job and determine which work is directly or indirectly related to cancer.

Finally, program surveys measured participants’ self-related judgment on their capability to pursue a doctoral degree and their perception of having the necessary skills to achieve this goal.

## Statistical Analysis

Descriptive statistics were performed for all variables. Categorical variables were presented as frequencies and percentages. Values derived from Likert scales were treated as continuous and means and standard deviations calculated. Wilcoxon signed rank tests were used to determine significant changes in participants’ academic self-efficacy and confidence before and after attending the summer institute. All statistical analysis was performed using SPSS version25 statistical software and *P* values of < 0.05 were considered statistically significant.

The authors have created an *Éxito!* Program replication manual that is viewable and downloadable on the *Éxito!* Website (www.exitotraining.org). The manual provides detailed information on replicating the *Éxito!* Program and provides downloadable copies of all survey instruments and program materials.

## Results

Between 2011 and 2015, *Éxito!* received 147 applications; 101 participants were selected and attended the *Éxito!* Summer institute.

### Participant Characteristics

At the time of acceptance into the program, the average participant age was 29 (IQR: 25 to 31 years). Most participants were female (87.1%), single (70.3%), with no children (85.1%), and US born (71.3%). More than half (58.4%) indicated that English was their primary language; the other 39.6% indicated that Spanish was their primary language. The majority of participants self-identified as Mexican (48.7%) or Puerto Rican (26.9%). In terms of educational attainment, 65% of *Éxito!* participants were enrolled in master’s programs at the time of attending the summer institute; 35% were master’s level graduates. Table 1 provides information on participant demographics and education characteristics.

**Table 1:** *Éxito!* summer institute participant demographics and educational characteristics (*n* = 101)

Variable	
Gender, *n* (%)	
Female	88 (87.1%)
Marital status, *n* (%)	
Single	71 (70.3%)
Married	21 (20.8%)
Other	9 (8.9%)
Children, *n* (%)^a^	
No	86 (85.1%)
Country of origin, *n* (%)^a^	
US born	79 (78.2%)
Outside US	20 (19.8%)
Mexico	10 (50.0%)
Dominican Republic, Guatemala, Colombia, Chile, Belize	10 (50.0%)
Primary language, *n* (%)	
English	59 (58.4%)
Location of residency, *n* (%)	
West Region	42 (41.2%)
Southwest Region	32 (31.5%)
Caribbean (Puerto Rico)	12 (11.8%)
Northeast Region	6 (5.7%)
Southeast Region	5 (4.6%)
Midwest Region	4 (3.9%)
Family expectations, *n* (%)	
Complete a bachelor’s degree	64 (63.3%)
Complete a master’s degree	23 (22.7%)
Complete a doctoral degree	14 (13.9%)
Financial assistance: loans for tuition and living expenses, *n* (%)^a^	
Yes	82 (81.2%)
Financial assistance: grants, scholarships, and/or fellowships, *n* (%)^a^	
Yes	84 (83.2%)
Father’s education, *n* (%)	
Grade school–high school	42 (41.7%)
Some college	20 (19.8%)
Bachelor’s degree	23 (22.8%)
Master’s degree	3 (3.0%)
Doctoral degree	7 (6.9%)
Do not know	4 (4.0%)
Mother’s education, *n* (%)	
Grade school–high school	42 (41.7%)
Some college	25 (24.8%)
Bachelor’s degree	24 (23.8%)
Master’s degree	4 (4.0%)
Doctoral degree	3 (3.0%)
Do not know	1 (1.0%)

Notes: Demographic data reflects participants who attended the 2011–2015 summer institutes

^a^Missing responses *n* = 2 (2%)

Year 1 of the program was a formative development period, which yielded modifications to the initial instruments provided by MTPCCR. Data from cohort 2011 were excluded from this analysis due to these changes. This report includes data for cohorts 2012–2015 (*n* = 77), all of which used the same evaluation instruments. Internships were offered in years 2 through 4. In year 5, funds were not available through the end of 6-month internship period due to the grant end date.

### Pre- and Post-Summer Institute Survey Results

Prior to the summer institute, participants completed a survey that assessed perceptions of barriers and facilitators and academic self-efficacy. Overall, 43.4% of respondents indicated that they were unsure, and 9.6% said they were not confident that they would apply to a doctoral program in the next year; 18.1% stated they were unsure they would apply in the next 5 years. Participants also indicated they were unsure (39.8%) they would pursue a career in cancer research.

After the summer institute, participants’ confidence about applying to a doctoral program in the next 5 years increased significantly (Table 2). Academic self-efficacy also improved significantly among all measured domains (Table 3).

**Table 2:** Participants’ average scores for confidence toward applying to a doctoral program and pursuing a career in cancer research before and after attending the *Éxito!* summer institute (*n* = 77)

	Pre-SI	Post-SI	*p* value^a^	
	M (SD)	M (SD)	Z	*p*
How confident are you that you will apply to a doctoral program in the next year?	3.48 (1.03)	3.56 (1.37)	0.82	0.41
How confident are you that you will apply to a doctoral program in the next five years?	4.32 (0.73)	4.55 (0.84)	2.67	0.01
How confident are you that you will pursue a career in cancer research?	3.83 (0.77)	3.91 (0.95)	1.10	0.27

**Table 3:** Participants’ average scores for academic self-efficacy before and after attending the *Éxito!* Summer Institute (*n* = 77)

	Pre-SI	Post-SI	*p* value^a^	
	M (SD)	M (SD)	Z	*p*
I have the skill to apply to a doctoral program.	4.03 (0.89)	4.44 (0.66)	3.47	0.00
I can get accepted into a doctoral program.	3.90 (0.90)	4.38 (0.71)	4.04	0.00
I can get accepted into a doctoral program of my choice.	3.39 (0.91)	4.00 (0.77)	4.59	0.00
I can write a strong personal statement.	3.76 (0.81)	4.25 (0.70)	4.63	0.00
I can obtain an acceptable GRE score.	3.58 (0.82)	3.99 (0.64)	4.16	0.00
I can accomplish my academic goals.	4.45 (0.60)	4.66 (0.50)	2.67	0.01
Financial challenges will not stop me from applying to a doctoral program.	2.91 (1.15)	4.18 (0.92)	6.42	0.00
I have adequate social support to pursue a doctoral degree.	4.08 (1.04)	4.35 (0.89)	2.61	0.01
My cultural background will serve as an asset in a doctoral program.	4.43 (0.80)	4.83 (0.44)	3.65	0.00

^a^Wilcoxon signed rank test used to assess significance

Response scale ranges from 1 to 5 (1 = strongly disagree to 5 = strongly agree).

The post-summer institute survey also evaluated participants’ experience during the summer institute. Most strongly agreed (68%) or agreed (29%) that the summer institute objectives met their needs; 83% strongly agreed and 15% agreed that the program motivated them to overcome barriers to obtaining a doctoral degree; and 80% strongly agreed and 15% agreed that the summer institute enhanced their overall self-confidence. Further, when responding to the question—“To what extent has the summer institute affected your interest in pursuing a doctoral degree?”—the response ranking scored very high (M = 9.08, SD = 1.46). Similarly, when responding to the question—“To what extent has the summer institute affected your interest in pursuing a career in cancer research?”—the mean score was M = 8.36 (SD = 1.72).

In the post-institute qualitative component of the survey, the vast majority of the participants’ open-ended responses lauded the program as very motivational and beneficial, as illustrated by the following comments:

The institute played a very important role in guiding my next steps towards doctoral school by exposing me to other Latinos who have overcome barriers and life challenges; showing me various research interest and perspectives worth pursuing in a doctoral program; and providing me with a network of students and professionals willing to serve as mentors.

The [Éxito! summer institute] reaffirmed my belief to apply to doctoral programs. It opened up my eyes to additional opportunities in cancer research that I may not have previously thought of. It was also a tremendous experience to be surrounded by people with your same interests and that motivated me even more to go into a career in cancer research.

### Internships

*Éxito!* awarded 21 internships from 2012 to 2014. Each project varied, with the majority of interns working on secondary data analysis (*n* = 15) using various cancer-related data sets and obesity-related data sets. Other projects included literature reviews (*n* = 4), community health education (*n* = 1), and original research (*n* = 1).

Based on findings from the pre- and post-internship surveys, interns’ research skills significantly improved across all measured domains (Table 4). Confidence to pursue a doctoral degree and/or career in research did not improve significantly; however, there were marked increases in pre-to-post confidence scores. Mentors of *Éxito!* interns (*n* = 19) were asked about their interns’ motivation to apply to a doctoral program at the start of the internship and after. Before starting, 47% of mentors indicated that their intern was highly motivated, 37% somewhat motivated, and 16% neither motivated nor unmotivated. After internships, mentors indicated that their intern was highly (63%) or somewhat (37%) motivated. Throughout the 5 years of the program, two mentors provided mentorship two different years, resulting in a number of mentors (*n* = 19) that is less than the number of interns (*n* = 21).

**Table 4:** Interns self-reported research skills before and after completing an Éxito! internship (*n* = 21)

	Pre-internship	Post-internship	Wilcoxon signed rank test	
	M (SD)	M (SD)	Z	*p*
Conducting literature reviews	3.48 (0.81)	4.33 (0.73)	3.35	0.00
Writing skills	3.08 (0.74)	3.90 (0.77)	3.63	0.00
Time management	3.67 (0.73)	4.14 (0.66)	2.49	0.01
Interpersonal skills	4.14 (0.73)	4.67 (0.58)	2.84	0.01
Computer skills	3.90 (0.70)	4.33 (0.66)	2.50	0.01
Statistical research	2.67 (1.16)	3.43 (1.08)	3.23	0.00
Conceptual skills	3.16 (0.83)	4.00 (0.82)	3.56	0.00

Response scale ranges from 1 to 5 (1 = strongly disagree to 5 = strongly agree).

### *Éxito!* Alumni Doctoral Application and Acceptance

As of December 2015, 43% of all alumni (*n* = 101) applied to doctoral programs and 29.7% (*n* = 30) are currently enrolled in a doctoral programs. Among the doctoral enrollees, 17% (*n* = 5) completed *Éxito!* internships. Further, 33.3% (*n* = 10) of currently enrolled/and accepted doctoral students indicated that their current job/research was directly related to cancer (e.g., worked focused on cancer screening, patient navigation, clinical trials) and 36.7% (*n* = 11) indicated their job indirectly related to cancer (e.g., worked focused on health promotion, health education, intervention research). Similarly, when given the statement that they would pursue a career directly related to cancer after obtaining a doctoral degree, 53.3% (*n* = 16) of doctoral students indicated they either strongly agreed (30%) or agreed (23.3%). In addition, survey results indicated that, among current master’s students and master’s graduates (*n* = 54), 25.9% (*n* = 14) indicated they were confident (or very confident) they would apply to a doctoral program in the next year and 59.3% (*n* = 32) of alumni in the next 5 years.

## Discussion

The *Éxito!* rate of doctoral program enrollment (30%) after 5 years is nearly double that for MTPCCR Latinos (17%) after 12 years [3]. *Éxito!* is able to address issues specific to Latino students and recognize the value of their culture, helping them to see their potential roles in eliminating disparities, and giving them the support and confidence they need to take the next academic step. Exposure to successful Latino role models and like-minded peers were powerful forms of verbal persuasion. In *Éxito!*, students found a “sense of belonging” and viewed themselves as a larger force rather than simply a “minority.”

The first 5 years of *Éxito!* succeeded in improving participants’ confidence in their ability and desire to pursue and complete doctoral-level degrees and careers in cancer control research that focus on Latino cancer health disparities. The program addressed many of the barriers that Latinos face in attempting to rise to the doctoral level and thus to achieve greater representation as leaders in public health. The program will continue to offer the summer institute and internships for 125 master’s level students and professionals between 2015 and 2020 with support from a second round of NCI funding. Throughout, we will continue to modify the program based on participant feedback.

The fact that *Éxito!* results far exceeded those of the multi-ethnic MTPCCR using the same model but primarily targeting and tailoring for Latinos supports the strength of role modeling and cultural concordance in both encouraging Latino students to apply by increasing their self-efficacy in a highly supportive environment, and enabling them to overcome a broad range of barriers. This includes increasing their efficacy in communicating their needs, applying for financial assistance, gaining acceptance in campus environment and how to identify and incorporate mentorship as well as peer support. Some notable lessons were learned in our first 5 years. Importantly, *Éxito!* alumni and doctoral students continue to need support after attending the summer institute related to issues such as the doctoral application process, insufficient mentoring for doctoral students, and managing course work during their doctoral program. Because *Éxito!* has been renewed for another 5 years, we are able to address these needs in the new funding cycle (2015–2020) by focusing on two important constructs: doctoral level persistence and career development.

Doctoral programs are described as consisting of three distinct stages: stage I is the first year of a doctoral program, stage II is the start of the second year through the completion of requirements for a doctoral degree, except for the dissertation, and stage III is the dissertation and defense [23, 24]. Previous studies have indicated that most attrition occurs during stage I and stage II [23, 24].

For *Éxito!* alumni currently enrolled in doctoral programs, we are developing a stage-based pipeline of support. We use social media outlets like Facebook and LinkedIn to encourage alumni to contact us so that we may provide further support and connect them with other alumni and current doctoral students who have faced similar challenges or concerns. These innovations will also help recent alumni connect with one another after attending the summer institute. This connection facilitates a more expansive network with individuals from similar cultural backgrounds who share concerns, challenges, and questions related to pursuing doctoral education. In addition, quarterly webinars are being presented on topics chosen in direct response to the needs voiced by current alumni, such as: (1) applying to a doctoral program (statement preparation, standardized test preparation, funding sources, etc.), (2) how to succeed in a doctoral program (selecting mentors, qualifying exams, dissertation preparation, etc.), and (3) general career development (careers in academia/research/private sector, CV preparation, scholarly writing for publication, grant writing, etc.). In addition, more tailored webinars will be offered for program alumni who are currently enrolled in doctoral programs. The content discussed during these webinars will be guided by the topics most important to the doctoral students.

Other lessons learned from *Éxito!* reflect what has been stated consistently in the literature: most *Éxito!* participants are the first in their family to pursue a higher education; among the most common educational barriers is the lack of adequate social support [10, 14, 25], and academic programs may offer little to no support, including few role models as mentors and peers. Indeed, ample evidence demonstrates the positive impacts of role models on students [26, 27].

Additionally, the majority of our *Éxito!* participants reported using loans and grants to fund their undergraduate and master’s degrees such that their greatest barrier to pursuit of a doctoral degree was the financial burden. Many students also lack the real-world experience and academic preparedness [14] needed to succeed in a doctoral program. For these reasons, our internships aimed to engage participants in research, expand their research skills, and show them the true value of evidence.

As the Latino population grows, Latino enrollment in post-secondary institutions will likely increase [12]. However, barriers such as those discussed here will influence Latino attrition rates, with the greatest disparity at the doctoral level [10, 14, 25, 28]. The *Éxito!* program stands as a robust example of a strategy to strengthen the pipeline for Latino representation at the doctoral level so that the next generation of health practitioners and researchers is representative of the US population and that culturally relevant solutions can more effectively reduce health disparities and improve health equity.

### Implications

The MTPCCR initially demonstrated the portability and replicability of its cancer control diversity pipeline program model to a second institution within California [19]. *Éxito!* has added another replication, demonstrating the model’s adaptability to a different region of the country and its effectiveness in targeting a specific population, Latinos/Hispanics. Indeed, *Éxito!*’s results exceeded those for Latinos in the MTPCCR. The successful adaptation of *Éxito!* from the MTPCCR program is evidence that the basic program components can be disseminated and adapted to fit the needs of other research institutions. After 5 years, the evidence of its acceptance and value among the Latino/Hispanic population show that the program should be ready for further replication at Hispanic Serving Institutions (HSIs) and beyond the Southwestern region.

## References

[1] Cancer facts & figures for Hispanics/Latinos 2012-2014 (2016) https://www.cancer.org/content/dam/cancer-org/research/cancer-facts-and-statistics/cancer-facts-and-figures-for-hispanics-and-latinos/cancer-facts-and-figures-for-hispanics-and-latinos-2012-2014.pdf

[2] Smith BD, Smith GL, Hurria A, Hortobagyi GN, Buchholz T (2009). Future of cancer incidence in the United States: burdens upon an aging, changing nation. J Clin Oncol.

[3] Pasick RJ, Kagawa-Singer M, Stewart SL, Pradhan A, Kidd SC (2012). The minority training program in Cancer control research: impact and outcome over 12 years. J Cancer Educ.

[4] Beckles GL, Truman BI, CDC (2011). Education and income --- United States, 2005 and 2009. Morb Mortal Wkly Rep.

[5] Liao Y, Bang D, Cosgrove S, Dulin R, Harris Z, Taylor A, Giles W (2011). Surveillance of health status in minority communities - racial and ethnic approaches to community health across the U.S. (REACH U.S.) risk factor survey, United States, 2009. MMWR Surveill Summ CDC.

[6] Singh GK, Hiatt RA (2006). Trends and disparities in socioeconomic and behavioural characteristics, life expectancy, and cause-specific mortality of native-born and foreign-born populations in the United States, 1979-2003. Int J Epidemiol.

[7] Davis JL, Ramos R, Rivera-Colón V, Escobar M, Palencia J, Grant CG, Green BL (2014). The Yo me cuido(®) program: addressing breast Cancer screening and prevention among Hispanic women. J Cancer Educ.

[8] George S, Duran N, Norris K (2014). A systematic review of barriers and facilitators to minority research participation among African Americans, Latinos, Asian Americans, and Pacific islanders. Am J Public Health.

[9] Rivera-Nieves J, Abreu MT (2013). A call for investment in education of US minorities in the 21(st) century. Gastroenterology.

[10] Association of Schools of Public Health (2011) Annual data report 2011. Retrieved from: https://depts.washington.edu/sphnet/wp-content/uploads/2013/06/FINAL_ASPH-Annual-Data-Report-2011.pdf. Accessed 18 August 2016

[11] Lopez M, Fry R (2013) Among recent high school grads, Hispanic college enrollment rate surpasses that of whites | pew research center. Retrieved from: http://www.pewresearch.org/fact-tank/2013/09/04/hispanic-college-enrollment-rate-surpasses-whites-for-the-first-time/. Accessed 18 August 2016

[12] Stepler R, Brown R (2015) 2014, Hispanics in the United States Statistical Portrait. Pew Hispanic Center. Retrieved from: http://www.pewhispanic.org/2016/04/19/2014-statistical-information-on-hispanics-in-united-states/. Accessed 1 November 2016

[13] Crisp Gloria, Nora Amaury (2009). Hispanic Student Success: Factors Influencing the Persistence and Transfer Decisions of Latino Community College Students Enrolled in Developmental Education. Research in Higher Education.

[14] DePass A, Chubin DE (2009). Understanding interventions that encourage minorities to pursue research careers*. – Building a Community of Research and Practice*.

[15] Gardner SK, Holley KA (2011). “Those invisible barriers are real”: the progression of first-generation students through doctoral education. Equity Excell Educ.

[16] Terenzini PT, Springer L, Yaeger PM, Pascarella ET, Nora A (1996). First-generation college students: characteristics, experiences, and cognitive development. Res High Educ.

[17] Tate KA, Fouad NA, Marks LR, Young G, Guzman E, Williams EG (2015). Underrepresented first-generation, low-income college students’ pursuit of a graduate education: investigating the influence of self-efficacy, coping efficacy, and family influence. J Career Assess.

[18] Pasick RJ, Otero-Sabogal R, Nacionales MCB, Banks PJ (2003). Increasing ethnic diversity in cancer control research: description and impact of a model training program. J Cancer Educ.

[19] Yancey AK, Kagawa-Singer M, Ratliff P, Valdez A, Jiménez L, Banks B, Stewart S, Roe KM, Pasick RJ (2006). Progress in the pipeline: replication of the minority training program in cancer control research. J Cancer Educ.

[20] Freire P (1998). Cultural action and conscientization. Harv Educ Rev.

[21] Bandura A (1995) Self-efficacy in changing societies. New York: Cambridge University Press

[22] Prochaska JO, Velicer WF (1997). The Transtheoretical model of health behavior change. Am J Health Promot.

[23] Bowen WG, Rudenstine NL (1992). In pursuit of the PhD.

[24] Vaquera G (2008). Testing theories of doctoral student persistence at a Hispanic serving institution. J Coll Stud Retent.

[25] Cerna OS, Pérez PA, Sáenz V (2009). Examining the precollege attributes and values of Latina/o bachelor's degree attainers. J Hisp High Educ.

[26] Arellano AR, Padilla AM (1996). Academic invulnerability among a select group of Latino university students. Hisp J Behav Sci.

[27] Zalaquett CP, Lopez AD (2006). Learning from the stories of successful undergraduate Latina/Latino students: the importance of mentoring. Mentor Tutor.

[28] Taylor AZ, Graham S (2007). An examination of the relationship between achievement values and perceptions of barriers among low-SES African American and Latino students. J Educ Psychol.

